# Residents’ perceptions of their own sadness - a qualitative study in Norwegian nursing homes

**DOI:** 10.1186/s12877-015-0019-y

**Published:** 2015-03-08

**Authors:** Kristina Riis Iden, Sabine Ruths, Stefan Hjørleifsson

**Affiliations:** Research Unit for General Practice, Uni Research Health, Bergen, Norway; Department of Global Public Health and Primary Care, University of Bergen, Bergen, Norway

**Keywords:** Sadness, Depression, Nursing homes, Frail elderly, Coping strategies, Medicalization

## Abstract

**Background:**

Mood symptoms are highly prevalent among frail old people residing in nursing homes. Systematic diagnostics of depression is scarce, and treatment is not always in accordance with best evidence. The distinction between non-pathological sadness and depression may be challenging, and we know little of the older peoples’ perspectives. The aim of this qualitative interview study was to explore residents’ perceptions of their own sadness.

**Methods:**

We performed individual, semi-structured interviews with twelve older people residing in nursing homes with no dementia. The interview guide comprised questions on what made the informants sad and what prevented sadness. We recorded, transcribed verbatim and analysed the interviews using systematic text condensation.

**Results:**

The interviews revealed three main themes. I. Decay and loss of agency. The informants perceived their sadness to be caused by loss of health and functional ability, reliance on long-term care, dysfunctional technical aids and poor care. II. Loneliness in the middle of the crowd. Loss of family and friends, and lack of conversations with staff members and fellow patients were also sources of sadness. III. Relating and identity. The informants kept sadness at bay through: acceptance and re-orientation to their current life situation, maintaining narratives about their identity and belonging, and religiosity.

**Conclusions:**

Nursing home nurses and doctors should identify and respond to sadness that is a rational response to manageable causes. Further, identifying and supporting residents’ resources and coping strategies is a salutogenetic approach that may alleviate sadness.

## Background

Sadness is probably inherent to human existence, although its occurrence and intensity differ and individuals and cultures express it in various ways. Sadness is an affect associated with various experiences and represents an internalizing response to whatever initiated it. Normal sadness is defined as context specific, its intensity corresponds to the reason for being sad, and sadness diminishes when its reason is brought to an end [1]. In contrast to sadness as a normal emotional reaction, sadness is also a core symptom of depression.

Most older people residing in nursing homes have complex health problems and severe functional impairment. Depression is prevalent [2,3], and contributes to increased morbidity and mortality [4-6]. The important task of distinguishing “meaningful sadness from counterproductive depression” [7] may be challenging for the following reasons: Social involvement is decreased due to loss of friends and family and a shrinking social network [8], the diagnostic cut-off is defined by convention [9], biological markers are lacking to resolve ambiguous cases, and finally, depression may have various clinical expressions [10]. Unfortunately, previous research in nursing homes suggests a scarcity of systematic diagnostics of depression [11]. It even appears that when an auxiliary nurse reports that an old person living in a nursing home appears to be sad, the skilled nurse forwards this message to the nursing home doctor who initiates treatment with antidepressants without even interviewing the person [12].

Frail older people residing in nursing homes need comprehensive patient-centred health services [13]. According to Dowrick, one should ask “why” and “what for” when people experience sadness, and try to talk about it with them [14]. Antonovsky introduced the concept of Sense of Coherence (SOC) to explain human coping resources [15]. People who possess a strong SOC are able to comprehend their present life situation, find it meaningful, and maintain a good quality of life in spite of health decline [16]. Previous research indicates that a strong SOC is associated with less depression in the general older population [17], and nursing home populations in particular [18,19].

Being specialists in family medicine with broad clinical experience, we share an interest in depression in older people, nursing home medicine and medicalization. The first and second authors are experienced nursing home doctors, and the third author is qualified in ethics and philosophy. The present study has been motivated by concerns about potentially harmful medicalization of inevitable processes during the final stage of life, which can undermine the person’s own coping resources. Poor diagnostic work with lack of regard for residents’ perspectives, may lead to an inappropriate diagnosis of depression and overtreatment with antidepressant drugs. Nursing home staff should use person-centred approaches to deal with non-pathological sadness as well as sadness comprised by a diagnosis of depression. To supplement the perspectives of doctors and nurses, residents’ perspectives can provide useful knowledge for improving the health-promoting approach of nursing home services.

The aim of this qualitative interview study was to explore residents’ perceptions of sadness. The phenomenon of sadness rather than the medical diagnosis of depression was the focus of this study. Because information about the informants’ diagnoses or medication could disturb our attention to sadness, we decided not to collect any background information about the informants’ mental health.

## Methods

In Norway, nursing homes constitute part of the primary health care system provided by the municipalities. About 40,000 citizens live in nursing homes, which corresponds to 18% of inhabitants 80 years and older. About 80% of the people in long-term care in nursing homes have dementia [20]. Due to the residents’ complex health problems and frailty, they need extensive care round the clock, as opposed to older people residing in care homes who are more self-reliant. Part-time employed general practitioners provide most of the medical services.

We conducted a qualitative study in 2012–2013 in Rogaland County, Norway. As we intended to explore the subjective perceptions of frail persons, individual and qualitative interviews were the appropriate approach. Four nursing homes were invited and agreed to participate. The institutions were similar in their size and organisation; three had public funding and one was private. The nursing homes did not have a faith basis. None of the authors had ever worked in the participating nursing homes. The first author informed the staff members about the study protocol through meetings and letters. Registered nurses recruited residents whom they perceived as sad; they could, for example, have a sad expression or an unhappy voice, weep easily or lack the ability to enjoy pleasant events. A purposive sample of twelve long-term care residents older than 80 years without moderate or severe dementia was included; eight informants were women. The recruiting nurse checked the informants’ medical record with regard to dementia. No health information about the informants was collected.

The first author performed semi-structured, individual interviews with the informants. Each interview lasted about 30 minutes. For one informant, the interview was performed on two different days. After informing the participants cautiously about the purpose of the study, KRI started each interview by asking “Do you feel sad?” Each informant gave an affirmative answer that was followed up by various questions to explore what the informants considered to be the causes of their sadness, and how they could avoid being sad. Further, they were encouraged to elaborate on topics they had introduced already and that seemed to be important to them.

The interviews were audio-recorded and transcribed verbatim. All three authors performed the analysis in collaboration and interpretations were negotiated until agreement was achieved. The method used was systematic text condensation, as described by Malterud [21]. We divided the analysis into four steps: (1) reading the transcripts to obtain an overall impression; (2) identifying units of meaning characterizing diverse features of sadness and coding for these; (3) abstracting the contents of each of the coded groups; and (4) generalizing the description and concepts regarding how patients perceive their sadness. We performed interim analysis after five interviews. When we had interviewed 12 informants, we decided that the interviews provided rich enough material to illuminate the informants’ perceptions of their sadness. The planning and performing of the study, as well as the analysis and presentation of the results comply with the RATS-guidelines for qualitative research [22].

### Ethics and approval

The Western Regional Committee for Medical and Health Research Ethics approved the study (2012/541). All informants provided written consent before the interviews were performed. We have removed or disguised all informants’ identifiers so the informants described cannot be identified.

## Results

The interviews revealed three main themes: I. Decay and loss of agency; II. Loneliness in the middle of the crowd; III. Reconciliation and identity.

### I. Decay and loss of agency

The informants expressed that sadness was caused by health problems, functional impairment, dependency on care, dysfunctional technical devices and aids, and poor care. Among other conditions, they most frequently and empathetically stated urinary incontinence and care related to that condition as being humiliating and a significant cause of sadness.

*“I am often sad because I have to wear diapers and need help to use the bathroom. But I don’t like it when my diapers are wet and I’m completely dependent on care.”* (Woman)

Thus the informants described how illness would disable them and cause discomfort, pain, immobilization and make them dependent on care and on decisions made by caregivers. Several informants told stories about toilet routines that included sadness and frustration about the use of diapers, or even technical devices such as a lift used for toileting because of paretic limbs. Sometimes sadness and resignation dominated, as when a male informant reflected that diapers used for convenience interfered with his dignity:

*“It is OK with diapers since I don’t have to get up in the middle of the night and I can sleep well. But on the other hand…it is beneath my dignity.”*

In other cases frustration dominated, as in the case of the male informant with paretic lower limbs who had experienced that his wishes were ignored:

*“You know they decided to use that lift against my will and ever since that day my life here has become less tolerable.”*

Other examples of dysfunctional aids and technical devices that would trigger sadness included a wheelchair that did not fit the user, a pillow being shaped incorrectly, or technical aids for use in the bathroom being either humiliating or not fit for the purpose.

*“It is of great importance for me that I am in a good sitting position, that the food is tasty and that I am able to smoke a cigarette. But if I am not sitting well, I feel sad… Some technical devices can spoil my day.”* (Man)

Examples of poor care leading to sadness included nurses being mean when the informant needed to go to the bathroom at night. However, one informant explained that once she had informed the head nurse about what happened, the harsh nurses became more kind. Other informants would adjust to poor care:

*“If the mean nurse is on duty, I don’t dare to call for help.”* (Woman)

### II. Loneliness in the middle of the crowd

Loss of family and friends, lack of meaningful communication with fellow residents, and staff members having little time for conversation were also sources of sadness. Many informants told that they felt lonely because they had lost their spouse or other family members. They expressed difficulty and frustration about not being able to talk with other residents, since most of them had dementia. Thus one informant described how she had to repeat the same information again and again to her fellow resident with dementia.

*“She asks me the same question: where is the train heading for? And I answer her, whereby she shortly after asks me the same question again. It really annoys me and makes me sad.”* (Woman)

Separation from his spouse was a source of deep sadness for this male informant:

*“We have been married for ages. Moving into nursing home was like getting a divorce. It was very sad, even though she visits me every day.”* (Male)

Many informants emphasized that they wanted to converse with the nurses, but perceived them as being preoccupied with providing bodily care, medications and meals.

*“The nurses are always in short of time. They don’t have time to talk with us. They have so many tasks to be carried out, and they can’t be blamed for that…the nurses are very kind, but I would have preferred they sat down and talked with us… but the times they are changing. Everything and everyone is in a rush.*” (Woman)

The nursing home doctors were mostly absent from the informants’ accounts, the only two exceptions being one informant explaining that she would speak with the doctor when somatically ill, and another informant relating an incident when the doctor misunderstood her, since he thought she was sad because of her husband’s recent death, whereas the real cause of her sadness was urinary incontinence.

*“I became sad when they told me I needed to use diapers. I was really sad. Then the doctor came to visit me, and I told him I was sad because of the incontinence and the diapers I had to wear. He replied “I realize you are sad since you just recently lost your husband“. So he thought this was the reason for being sad.”* (Woman)

### III. Reconciliation and identity

According to the participants, what helped them avoid sadness was accepting the realities of old age, gratitude for remaining function and for care, relating to own and family’s life history, and religious activity. During the interviews, the informants actively engaged in viewing life in a larger context, demonstrating that they were still able to tell meaningful stories about their own lives and those of their families. Own achievements and dispositions in the past were reported – with obvious pride by some, but more often with a matter-of-factness, as were the doings of younger kin. In the informants’ narratives, past and current history established their identity in ways that conveyed an understanding of how to avert sadness. Sometimes the informants explicitly explained how past experiences were relevant even today:

*“For I must say, I have experienced two wars. Because when you are ninety-four, you have to accept a couple of things. … I have to accept being here since I need help. … I’m worn out. I could wish it was like when I was young, but that doesn’t help. But I’m not the kind who gives up.”* (Woman)

A couple of informants described how they initially felt grief and sadness when experiencing functional impairment, but after accepting the inevitability of impairment and dependence, they rather felt gratitude for their remaining level of function and for life itself.

*“At my age, I just have to accept the health decline. So I don’t spend much time reflecting on this.”* (Woman)

They also reflected on the importance of coping positively with declining health to avoid sadness:

*“We cope with life in different ways. Some complain about everything. Others don’t complain at all. For me…considering the circumstances… I have a good life here.”* (Woman)

Narratives about past achievements and family could also stand more alone, with more oblique references to their relevance for the topic of the interviews. Thus one informant proudly told about a sport badge he was awarded many years ago, another explained about his earlier profession and his granddaughter’s current career.

Some informants declared their faith, explained about their religious activities in the past, and conveyed that religious beliefs and practice still were a source of comfort to them. A woman expressed how important religion was to her as it had been for her late husband, treasuring the words and prayers of the vicar who visited her regularly. Some informants would have appreciated more religious meetings and activities in the nursing home. But praying was a good alternative:

*“I tell myself that I mustn’t be sad. I say a silent prayer. It makes me calm.”* (Woman)

## Discussion

When asked “why” and “how” they perceived their sadness, our informants answered in terms of health-related issues and poor care interfering with their dignity and agency, loneliness due to loss of kin and friends, and lack of communication with staff. But they also reported keeping sadness at bay through acceptance and re-orientation to their current life situation, through maintaining past and current narratives about their identity and belonging, and through religiosity.

### Strengths and limitations

In this study, an experienced nursing home doctor performed the interviews in the informants’ nursing homes, seeking to establish a safe atmosphere for sharing personal experiences. Because the informants were frail, individual interviews were probably the qualitative research method of choice. Recruitment performed by skilled nurses according to predefined criteria ensured the inclusion of informants who provided valuable information on the issue we wanted to explore. Selection bias because of voluntary study participation or the recruiting nurses’ preferences cannot be excluded though.

The informants had received written and oral information about the study in advance, and knew that the topic of the interview was sadness. They were obviously prepared for the interview and most informants had much they wanted to convey. Each interview lasted 15–30 minutes and was performed by a doctor-researcher who was a complete stranger to the informants. The interviewer sought to pursue any topics the informants shared, based on the idea that whatever they related might be relevant to their experience of sadness. Nevertheless, she may have missed some clues or have failed to pursue some topics appropriately. Although they talked openly about loss of bladder control and other private issues, the informants may have considered existential perspectives of life and death to be irrelevant topics to discuss with a doctor researcher. It would be interesting to determine whether longer or repeated interviews, interviews performed by a priest or someone with whom the informants already had a trusting relationship would yield different results regarding existential issues, but this could not be explored with the study design chosen.

As stated in the introduction, no information was collected about whether the informants were suffering from mental illness or received medications. Knowledge about a diagnosis of depression could have influenced the researchers’ preconceptions and interpretations of the interviews, and could even have introduced a bias in the conduction of the interviews themselves. Theoretically, all or none of the informants may have been suffering from depression, or anything in between. The following discussion concerns issues that are of relevance in any case.

### Theme I: Decay and loss of agency

Our findings suggest that health problems, functional impairment and poor care are major causes of sadness to older people residing in nursing homes, with urinary incontinence (UI) as the most empathetically stated example. This finding is in accordance with a large epidemiologic study in US nursing homes reporting that patients with UI experienced lower quality of life in the domains of dignity, autonomy and mood [23], and a study in Australian nursing homes and hostels showing that loss of dignity and independence caused by UI was strongly associated with quality of life [24]. An interview-study with older female residents residing in nursing homes with UI in Canada indicated that they felt regression to childhood and embarrassment, which led to loss of control, dignity, independence, and decision-making power [25]. This should raise concern because UI is a prevalent health condition affecting 67% of older women living in nursing homes [23]. We consider sadness a rational response to distress caused by UI and other health conditions. Therefore, more consideration should be given to understanding the lived experience from residents’ perspectives. This may inform and facilitate changes toward individualized care, empowerment of those experiencing UI, and improving specific health care education [25].

### Theme II: Loneliness in the middle of the crowd

Human life is meaningful when there is interaction with others in a social context, sharing and responding to each other’s experiences and thoughts [26]. In a systematic review of qualitative studies on living well in various types of care homes, connectedness with others and caring practices emerged as two out of four key themes [27]. The review, however, highlighted care home residents’ concerns about difficulty in forming appropriate relationships with others. This finding corresponds with the results of the present study; our informants voiced loneliness due to poor communication with their fellow residents with dementia, and limited contact with family, friends and nurses. This is relevant because loneliness, lack of social support and perceived inadequacy of care among older people residing in nursing homes were found to be associated with depression [28] and mortality [29]. Drageset *et al.* have shown that 56% of people without dementia living in Norwegian nursing homes experienced loneliness. Emotional closeness to significant others had even more impact than frequency of contact with family and friends [30]. They therefore advise that nurses should pay attention to emotional loneliness among nursing homes residents and especially give attention to the importance of having a close confidant who provides emotional support. Nursing homes in Norway have established special dementia care units for about 30% of institutionalized older people with dementia [31]. One might wonder, however, if corresponding initiatives should also be established for those without cognitive impairment to support their quality of life and functional ability.

### Theme III: Reconciliation and identity

In the face of challenging life events, people act differently depending on their coping strategies. In Antonovsky’s theory of salutogenesis, this coping capacity is defined as comprising a sense of coherence (SOC): a global orientation that expresses the extent to which one has a “pervasive, enduring though dynamic feeling of confidence”, a feeling which derives from experiencing one’s situation as meaningful, understandable and manageable [15]. A broad range of research has demonstrated a positive relationship between SOC and health, and probably also a positive relationship between SOC and future health [32]. Antonovsky anticipated that his theory on coping would specifically gain doctors working in primary care and geriatrics to understand and help people better by identifying and promoting elements that would enhance their SOC [15]. There is some evidence about how the SOC of older people residing in nursing homes can be supported, including the finding that social attachment was associated with a rise in SOC among residents without cognitive impairment during a five year follow-up [33].

Our findings regarding strategies for keeping sadness away can be interpreted in the light of Antonovsky’s theory. The informants’ reports about gratitude for care and acceptance of decline in old age, keeping in touch with family, narrating stories about their own identity, and engaging in religious activity indicate that the informants endeavour to re-create meaning, make sense of their predicament and maintain agency. This will render their situation meaningful, understandable and manageable and thus enhance their sense of coherence. Nursing home staff members should encourage all kinds of initiatives that may strengthen residents’ coping resources, and this should include communicating with residents to reduce their loneliness. This is in concordance with a previous qualitative study in two Norwegian nursing homes where Bergland and Kirkevold through interviews and participant observation found that the residents’ own attitude to their life situation and living in a nursing home was the single most important factor for their thriving [26]. Furthermore, in a qualitative study in Iceland, Hjaltadottir and Gustafsdottir found that people residing in nursing homes considered being recognized as a person with a family history and a meaningful life was crucial for their quality of life [34]. This indicates that nursing home staff members should encourage all kinds of initiatives that may strengthen residents’ coping resources, and this should include communicating with residents to reduce their loneliness and confirm their identity.

Some informants expressed wishes for religious meetings and worships. According to Pargament *et al*., religiosity provides meaning and confidence for older people [35]. Bosworth *et al*. have shown that positive religious coping had favourable effects on depression among older residents [36]. Norway is a highly secular society, and this is reflected by the organization of long-term care by the municipalities; i.e. most nursing homes do not have a faith basis. Consequently, residents’ needs for religious worship may be unfulfilled. Guidelines have been developed for many somatic diseases and nursing situations; the lack of corresponding initiatives with regard to spiritual needs may reflect underestimation and neglect of faith, including its meaning as a coping resource. Regardless of the religious foundation or lack of such in the nursing home, the importance of religion for many residents should be acknowledged.

### Implications for clinical practice and further research

The concern that motivated this study was that normal responses to inevitable processes towards the end of life may be inappropriately medicalized in nursing homes, contributing to suboptimal medical treatment and care, and undermining of the older people’s own resources. Our findings regarding sadness as a rational response to health problems, functional impairment, loneliness and poor care align with previous research on how institutionalized older people perceive their situation and what they value, highlighting priorities for patient-centred care [37]. Further, identifying and supporting residents’ resources and coping strategies is a salutogenetic approach that may alleviate sadness. When a resident in a nursing home appears to be sad, it seems that staff should respond by engaging with the resident to establish the cause of sadness, provide company and encourage contact with relevant others, support the resident’s own coping resources, as well as addressing any care-, diagnostic- and treatment issues ranging from e.g. urinary incontinence to the possibility of the resident being depressed.

Because sadness and depression are prevalent and partly overlapping conditions among older people residing in nursing homes, future studies should examine their boundaries, misclassification, associations with clinical and institutional characteristics, as well as the impact of depression on residents’ experience of sadness. Further, clinical studies are needed to examine the effects of different care- and therapeutic approaches on these conditions.

## Conclusion

Nursing home nurses and doctors should identify and respond to sadness that is a rational response to manageable causes. Further, identifying and supporting residents’ resources and coping strategies is a salutogenic approach that may alleviate sadness.

## References

[1] Horwitz AV, Wakefield JC (2007). The Loss of Sadness: How Psychiatry Transformed Normal Sorrow Into Depressive Disorder.

[2] Djernes JK (2006). Prevalence and predictors of depression in populations of elderly: a review. Acta Psychiatr Scand.

[3] Anstey KJ, von Sanden C, Sargent-Cox K, Luszcz MA (2007). Prevalence and risk factors for depression in a longitudinal, population-based study including individuals in the community and residential care. Am J Geriatr Psychiatry.

[4] Barca ML, Selbaek G, Laks J, Engedal K (2009). Factors associated with depression in Norwegian nursing homes. Int J Geriatr Psychiatry.

[5] Gonzalez-Salvador T, Lyketsos CG, Baker A, Hovanec L, Roques C, Brandt J (2000). Quality of life in dementia patients in long-term care. Int J Geriatr Psychiatry.

[6] Pot AM, Deeg DJ, Twisk JW, Beekman AT, Zarit SH (2005). The longitudinal relationship between the use of long-term care and depressive symptoms in older adults. Gerontologist.

[7] Lidz CW, Parker LS (2010). Issues of ethics and identity in diagnosis of late life depression. Ethics Behav.

[8] Mojtabai R (2014). Diagnosing depression in older adults in primary care. N Engl J Med.

[9] Maj M (2011). When does depression become a mental disorder?. Br J Psychiatry.

[10] Belmaker RH, Agam G (2008). Major depressive disorder. N Engl J Med.

[11] Iden KR, Engedal K, Hjorleifsson S, Ruths S (2013). Prevalence of depression among recently admitted long-term care patients in Norwegian Nursing Homes: Associations with diagnostic workup and use of antidepressants. Dement Geriatr Cogn Disord.

[12] Iden KR, Hjorleifsson S, Ruths S (2011). Treatment decisions on antidepressants in nursing homes: a qualitative study. Scand J Prim Health Care.

[13] Goodman C, Amador S, Elmore N, Machen I, Mathie E (2013). Preferences and priorities for ongoing and end-of-life care: a qualitative study of older people with dementia resident in care homes. Int J Nurs Stud.

[14] Dowrick C (2004). Beyond Depression : A New Approach to Understanding and Management.

[15] Antonovsky A (1987). Unraveling the Mystery of Health. How People Manage Stress and Stay Well.

[16] Lindstrom B, Eriksson M (2005). Salutogenesis. J Epidemiol Community Health.

[17] Eriksson M, Lindstrom B (2006). Antonovsky’s sense of coherence scale and the relation with health: a systematic review. J Epidemiol Community Health.

[18] Bjorklof GH, Engedal K, Selbaek G, Kouwenhoven SE, Helvik AS (2013). Coping and depression in old age: a literature review. Dement Geriatr Cogn Disord.

[19] Drageset J, Espehaug B, Kirkevold M (2012). The impact of depression and sense of coherence on emotional and social loneliness among nursing home residents without cognitive impairment - a questionnaire survey. J Clin Nurs.

[20] Selbaek G, Kirkevold O, Engedal K (2007). The prevalence of psychiatric symptoms and behavioural disturbances and the use of psychotropic drugs in Norwegian nursing homes. Int J Geriatr Psychiatry.

[21] Malterud K (2012). Systematic text condensation: a strategy for qualitative analysis. Scand J Public Health.

[22] Clark JP, Godlee FJT (2003). How to Peer Review A Qualitative Manuscript. Peer Review in Health Sciences.

[23] Xu D, Kane RL (2013). Effect of urinary incontinence on older nursing home residents’ self-reported quality of life. J Am Geriatr Soc.

[24] Sitoh YY, Lau TC, Zochling J, Schwarz J, Chen JS, March LM (2005). Determinants of health-related quality of life in institutionalised older persons in northern Sydney. Intern Med J.

[25] MacDonald CD, Butler L (2007). Silent no more: elderly women’s stories of living with urinary incontinence in long-term care. J Gerontol Nurs.

[26] Bergland A, Kirkevold M (2006). Thriving in nursing homes in Norway: contributing aspects described by residents. Int J Nurs Stud.

[27] Bradshaw SA, Playford ED, Riazi A (2012). Living well in care homes: a systematic review of qualitative studies. Age Ageing.

[28] Jongenelis K, Pot AM, Eisses AM, Beekman AT, Kluiter H, Ribbe MW (2004). Prevalence and risk indicators of depression in elderly nursing home patients: the AGED study. J Affect Disord.

[29] Drageset J, Eide GE, Kirkevold M, Ranhoff AH (2013). Emotional loneliness is associated with mortality among mentally intact nursing home residents with and without cancer: a five-year follow-up study. J Clin Nurs.

[30] Drageset J, Kirkevold M, Espehaug B (2011). Loneliness and social support among nursing home residents without cognitive impairment: a questionnaire survey. Int J Nurs Stud.

[31] Nokkeltall for helsesektoren. http://sites.helsedirektoratet.no/sites/nokkeltall/Sider/default.aspx. Accessed 18 February 2015

[32] Eriksson M, Lindstrom B (2005). Validity of Antonovsky’s sense of coherence scale: a systematic review. J Epidemiol Community Health.

[33] Drageset J, Espehaug B, Hallberg IR, Natvig GK (2014). Sense of coherence among cognitively intact nursing home residents–a five-year longitudinal study. Aging Ment Health.

[34] Hjaltadottir I, Gustafsdottir M (2007). Quality of life in nursing homes: perception of physically frail elderly residents. Scand J Caring Sci.

[35] Pargament KI, Smith BW, Koenig HG, Perez L (1998). Patterns of positive and negative religious coping with major life stressors. J Sci Study Relig.

[36] Bosworth HB, Park KS, McQuoid DR, Hays JC, Steffens DC (2003). The impact of religious practice and religious coping on geriatric depression. Int J Geriatr Psychiatry.

[37] Mathie E, Goodman C, Crang C, Froggatt K, Iliffe S, Manthorpe J (2012). An uncertain future: the unchanging views of care home residents about living and dying. Palliat Med.

